# COVID-19 related familial economic disruptions and eating disorder patients’ mental health concerns and motivation to recover

**DOI:** 10.1186/s40337-022-00709-4

**Published:** 2022-12-20

**Authors:** Monique Santoso, Carly E. Milliren, Elizabeth R. Woods, Sara F. Forman, Tracy K. Richmond

**Affiliations:** 1 Division of Adolescent/Young Adult Medicine, Boston Children’s Hospital, Boston, USA; 2 Institutional Centers for Clinical and Translational Research, Boston Children’s Hospital, Boston, USA; 3 Department of Pediatrics, Harvard Medical School, Boston, USA

**Keywords:** COVID-19, Eating disorders, Mental health, Comorbidity, Financial stress, Adolescents, Anorexia nervosa, Atypical anorexia nervosa

## Abstract

**Background:**

Family support has been shown to be important for adolescents and young adults (AYA) in eating disorder (ED) treatment. Many families were impacted by the pandemic, potentially altering their ability to support individuals in ED treatment. This study examined the association of COVID-19 related familial economic change with self-reported mental health (MH) and ED concerns in AYA seeking treatment for ED.

**Methods:**

AYA patients with EDs aged 10–27 years enrolled in the Registry of Eating Disorders and their Co-morbidities OVER time in Youth (RECOVERY) completed an additional COVID-19-specific survey (*n* = 89) that assessed their perception of the effects of the pandemic on their lives and their ED. Participants self-reported on familial economic disruptions, measured through a composite score of four markers: (1) family member’s work hours cut, (2) family member was required to stop working, (3) family member lost job permanently, and (4) family lost health insurance/benefits. In bivariate analyses, we examined the association between self-reporting any familial economic disruption and self-reported changes in intrusive ED thoughts, feelings of anxiety, feelings of depression, feelings of isolation, and motivation to recover from their ED. Logistic regression models were used to examine the association between familial economic disruptions on self-reported changes in ED/MH affect and motivation to recover adjusting for age and ED diagnosis.

**Results:**

Forty-six percent of participants self-reported that the pandemic had resulted in at least one economic familial disruption. Of patients reporting any familial economic disruption, 29% reported decreased motivation for ED recovery, and over 75% reported worsening feelings of depression, anxiety, isolation, and/or intrusive eating disorder thoughts. Reporting any COVID-19 familial economic disruption was marginally associated with feelings of isolation (*p* = 0.05). Though the findings were only marginally significant, the odds of reporting worsening feelings of depression, anxiety, intrusive ED thoughts or motivation to recover were nearly twice in those who reported a COVID-19-related familial economic disruption compared to those who did not report such a disruption.

**Conclusions:**

Family-related economic disruptions are associated with ED/MH-related concerns and motivation to recover from an ED during the COVID-19 pandemic in AYA patients.

## Plain English summary

Following the adoption of stay-at-home regulations to curb the spread of the COVID-19 pandemic, children’s hospitals and emergency rooms across the United States saw a tremendous uptick in adolescents and young adults with eating disorders (ED) and mental health (MH) concerns. COVID-19 related regulations had economic consequences, with family members reduction in work hours, temporary or permanent termination from work, and/or loss of their health insurance or benefits. This study sheds light on the association between COVID-19 related familial economic disruptions and mental health and eating disorders (MH/ED) concerns and motivation to recover from an ED in patients receiving care for ED (89 participants). Of patients reporting some economic familial disruption, three-fourths self-reported worsening mental health (i.e, worsening feelings of depression, anxiety, isolation, or intrusive eating disorder thoughts). Patients who perceived familial economic disruption due to the COVID-19 pandemic had almost a two-fold increased likelihood of worsening eating disorders and mental health (ED/MH) concerns and/or motivation to recover. Our study underscores the need to consider the economic effects of the pandemic on the well-being of adolescents/young-adults with ED, particularly those from financially vulnerable families.

## Background

Lockdown and shelter-in-place regulations adopted to control the spread of COVID-19 in early 2020 upended the lives of all and shed light on the existing health inequities that existed among vulnerable populations [1]. The elderly, infants and children, individuals with underlying physical and mental health conditions, persons with disabilities, pregnant women, persons institutionalized or without a home were most at risk for loss and worsening quality of life [2]. Furthermore, essential workers and medical professionals, who were required to work despite lockdown and shelter-in-place regulations, were also at increased psychological risk [3]. Among those affected were adolescents.

Not surprising, many adolescents experienced negative psychological consequences of the pandemic. Patients with eating disorders were particularly impacted [1]. The disruptions in daily life, reported increase in triggering environments, changes in family functioning, and decreased access to healthcare may all have contributed to individuals with eating disorders experiencing a worsening of their condition [2–5]. There has been a corresponding uptick in eating disorders related inpatient admissions, hospital bed-days, outpatient care-related inquiries, and emergency department visits post-pandemic compared to pre-pandemic globally. Therefore, it is critical to understand particular COVID-19 related factors that can be addressed to reduce further burden to individuals, families, and hospital systems [6–11].

Moreover, the pandemic had variable effects on patients with different types of eating disorders with some studies showing negative and others showing positive pandemic-related effects on EDs [23–25]. A systematic review on the studies investigating the effects of the COVID-19 pandemic on ED by Devoe et al. (2021) has indicated that patients diagnosed with anorexia nervosa (AN) and bulimia nervosa (BN) did not present with significant weight-or BMI-related changes while those with binge-eating disorder (BED) and BN reported more frequent binge eating episodes during the COVID-19 pandemic. [23] Patients with AN and BN were found to have improved ED symptomology with improvement in BMI for patients with AN due to active treatment involvement during the pandemic. [23] This review has underscored the large extent of studies investigating the influence of COVID-19 related disruptions on patients with mental health (MH) and eating disorders (ED) conditions. Another review by Haghshomar et al. (2022) has indicated that individuals with pre-existing mental health conditions such as EDs were more vulnerable to COVID-19 related changes, creating increased risk for anxiety, depression and insomnia symptomology, compared to those without pre-existing mental health conditions [24].

Prior research has demonstrated that family support is important to aid ED treatment outcomes and greater likelihood of patient recovery [12, 13]. Additionally, the alleviation of stress on the family unit has been shown to be an important factor in the success of ED-related treatment and recovery [12]. Nonetheless, the COVID-19 pandemic has led to increased economic burden and thus stress on families due to factors such as a decrease in working hours, loss of job, or loss of insurance. The National Bureau of Labor Statistics (2020) reported 1 in 4 people having to transition to telework or work from home for pay and over 31.3 million who quit their jobs or engaged in fewer work hours due to loss of employer business/closures during the early months of the COVID-19 pandemic [14]. Families experiencing such loss or disruptions with the accompanying stress may have less capacity to support family members with EDs and thus, may unintentionally contribute to worse outcomes [15].

Understanding how COVID-19 related familial economic burdens are associated with ED-related outcomes is important to further ED prevention and intervention efforts. To address these gaps in the literature, we set out to examine the association of participant-reported COVID-19 related familial economic disruption with participant-reported ED and mental health (MH) concerns. We hypothesized that participants who reported greater economic consequences of COVID-19 would have higher eating disorders and mental health (ED/MH) concerns and lower motivation to recover from an ED diagnosis than those who reported less economic consequences of COVID-19.

## Methods

### Study sample

Our study sample (*n* = 89) was made up of a subset of adolescent and young adult participants enrolled in the Registry of Eating Disorders and their Co-morbidities OVER time in Youth (RECOVERY) who completed an additional COVID-19-specific survey. The RECOVERY study is a longitudinal registry of patients with EDs seeking care in the outpatient ED program at Boston Children’s Hospital (BCH). RECOVERY uses web-based surveys every 3 months in the first year of participation, followed by 6 months thereafter. Participants were recruited between June 2017 and August 2020. In light of the COVID-19 pandemic, during July 2020, the RECOVERY participants were asked to complete an additional COVID-19 related survey on their perceived impact of the pandemic on their social and economic lives, ED treatment, ED/MH related symptomology and behaviors, and overall general wellbeing. Fifty-six percent of RECOVERY participants responded to this survey that was sent off-cycle for scheduled RECOVERY questionnaires, and without the typical remuneration offered. Of the participants in our study, 4.5% reported living alone (*n* = 4) and 95.5% (*n* = 85) reported not living alone in a binary measure of living alone or not during the pandemic. The RECOVERY study and this survey were approved by the BCH Institutional Review Board.

### Survey measures

Demographic patient characteristics were obtained from participants’ baseline RECOVERY survey responses. Measures of the COVID-19 survey were developed by researchers at the University Of North Carolina Centre Of Excellence for Eating Disorders (NCEED) [22].

### Primary predictor variables

*COVID-19 related familial economic disruption* Participants self-reported on the COVID-19 related familial economic disruption, measured through a composite score of the following four markers:

1. Cut back hours at work: “A member of my family had to cut back hours at work.” (Answer Choices: Yes/No).
2. Temporary work stoppage: “A member of my family was required to stop working (expect to be called back).” (Answer Choices: Yes/No).
3. Permanent job loss: “A member of my family lost their job permanently.” (Answer Choices: Yes/No).
4. Health insurance/benefits loss: “My family lost health insurance/benefits.” (Answer Choices: Yes/No)

Responses (Yes = 1, No = 0) from the four questions were added to give a score between 0 and 4 and collapsed into any ($\ge$ 1) vs. no (0) impact.

### Primary outcomes

Participants were asked to rate, on a 5-point Likert scale from “increased significantly” to “decreased significantly”: “How has the COVID-19 pandemic affected each of the following:” “Feelings of anxiety,” “Feelings of depression,””Feelings of isolation,” “Intrusive ED thoughts,” and “Motivation to recover from an ED.” We then categorized the responses into increased (increased significantly, increased somewhat), no effect, and decreased (decrease somewhat, decreased significantly). These were further collapsed into dichotomous variables for worsening vs. no change/improving for adjusted analysis. An increase in feelings of depression, anxiety, isolation or intrusive thoughts, or a decrease in motivation to recover were considered “worsening.”

### Additional variables

*Age* Participant age at the time of COVID-19 survey completion was calculated using the date of birth obtained from the RECOVERY study’s baseline survey and date of the COVID-19 survey.

*ED Diagnosis* Participants self-reported their ED diagnosis in the COVID-19 survey by answering the question: “Which of the following EDs do you currently have or have you had in the past? (Please check all that apply)” and were given a list of eight options: (1) anorexia nervosa (AN), (2) atypical anorexia nervosa (AAN), (3) avoidant restrictive food intake disorder (ARFID), (4) bulimia nervosa (BN), (5) binge-eating disorder (BED), (6) purging disorder, (7) other eating issue(s)/disorder(s), and (8) I don’t know/Unsure.

*Race/ethnicity* Our baseline survey included a question that asked participants to select all that applied from the following options: Hispanic/Non-Hispanic, American Indian or Alaska Native, Asian, Black or African American, Middle Eastern/North African, Native Hawaiian or other Pacific Islander, White/Caucasian or another race. We constructed a mutually exclusive race/ethnicity variable consisting of non-Hispanic white, Asian, Hispanic, Multiracial, non-Hispanic Black or African-American, and Other race.

*Sex* Participants self-reported their sex assigned at birth (female, male, or another sex) in the RECOVERY baseline survey.

*Length of current treatment* Length of time participant had engaged in current ED treatment was calculated from the date of the intake in the outpatient ED program from which the participants were recruited to the date of the COVID-19 survey completion.

### Statistical analysis

We examined frequencies (percent) for categorical variables and means (standard deviations) for continuous variables. To examine potential response biases, RECOVERY participants who responded to the COVID-19 survey were compared to non-respondents on demographic factors (age, race/ethnicity, and sex) and ED diagnosis using t-tests for continuous variables and *χ*^2^ tests for categorical variables. We examined bivariate associations between reported pandemic-related familial economic impact and participant-reported changes in intrusive ED thoughts, feelings of depression, anxiety, isolation, and motivation to recover from ED using *χ*^2^ tests (or Fisher’s exact test where appropriate). Logistic regression models were used to examine the association between pandemic-related familial economic impact and self-reported worsening in ED/MH concerns (increase vs. no change/decrease) and motivation to recover (decrease vs. no change/increase), adjusting for age and ED diagnosis. All analyses were conducted using SAS (v9.4; Cary, NC) with *p* < 0.05 considered a statistically significant result.

## Results

For this study, 56% (*n* = 89) of RECOVERY participants responded. Respondents did not differ from the overall RECOVERY participant sample on age at enrollment, ED diagnosis, race/ethnicity, or sex.

Table 1 illustrates the demographic and clinical characteristics of our sample. Restrictive EDs (84%) were the most common diagnoses. The average age of the participants at survey completion was 18.9 years. Our survey had largely female respondents (89%) who identified as White, and non-Hispanic (78%). Of these, 84% were diagnosed with anorexia nervosa (AN) and atypical anorexia nervosa (AAN). More than half of the participants had been in treatment for 2 years or more (53%).

**Table 1:** Demographic characteristics by self-reported pandemic-related economic consequences among COVID-19 RECOVERY study survey respondents (*n* = 89)

	*n* (%)			*p*-value
	Overall (*n* = 89)	Pandemic-related economic consequences		
		Any (*n* = 41)	None (*n* = 48)	
Age at survey completion (years), mean (SD)	18.9 (2.9)	19.2 (3.2)	18.7 (2.7)	0.43
Age 18 or above at survey completion	56 (63%)	26 (63%)	30 (63%)	0.93
Female at birth	80 (89%)	39 (95%)	41 (85%)	0.17
Race/Ethnicity				
White, non-Hispanic	69 (78%)	31 (76%)	38 (79%)	0.69
Other race/ethnicity^a^	20 (22%)	10 (24%)	10 (21%)	
Restrictive Eating Disorder Diagnosis	75 (84%)	34 (83%)	41 (85%)	0.75
Length of Eating Disorder Treatment				0.49
< 1 year	8 (9%)	2 (5%)	6 (13%)	
1–2 years	34 (38%)	16 (39%)	18 (38%)	
2 years or more	47 (53%)	23 (56%)	24 (50%)	

^a^ Other race comprised of n = 7 Asian, *n* = 6 Multiracial, *n* = 4 Hispanic, *n* = 2 Other race and *n* = 1 Black

*RECOVERY* Registry of eating disorders and their Co-morbidities OVER time, *SD* Standard deviation

Among the 89 respondents, 46% reported at least one familial economic disruption associated with the pandemic. To this end, 28% of participants reported one impact, and 18% reported two or more impacts. Familial economic disruptions reported by participants included a family member having to cut back hours at work (37%), a family member was required to stop working temporarily (17%), a family member permanently lost a job (12%), family lost health insurance (3%).

Our sensitivity analysis for participants with AN/AAN diagnoses illustrated that the socioeconomic breakdown of participants with this diagnosis is similar to the full sample with a mean age of 19.1 years with 67% over 18, 79% identifying as white and 92% indicating female sex-at-birth. Of these, 45% reported any familial economic disruption.

On the impact of the COVID-19 pandemic on MH/ED concerns, 29% of respondents reported that the COVID-19 pandemic decreased their motivation to recover from ED, 73% reported increased feelings of depression, 77% reported increased feelings of anxiety, 80% reported increased feelings of isolation, and 74% reported increased intrusive ED thoughts (Table 2).

**Table 2:** Association of self-reported pandemic-related economic consequences and change in eating disorder/mental health concerns (*n* = 89)

	*n* (%)			*p*-value
	Overall (*n* = 89)	Pandemic-related economic consequences		
		Any (*n* = 41)	None (*n* = 48)	
Change in motivation to recover from an eating disorder				0.27
Decreased	26 (29%)	15 (37%)	11 (23%)	
No effect	40 (45%)	15 (37%)	25 (52%)	
Increased	23 (26%)	11 (27%)	12 (25%)	
Change in feelings of depression				0.19
Decreased	4 (5%)	3 (7%)	1 (2%)	
No effect	20 (22%)	6 (15%)	14 (29%)	
Increased	65 (73%)	32 (78%)	33 (69%)	
Change in feelings of anxiety				0.72
Decreased	2 (2%)	1 (2%)	1 (2%)	
No effect	19 (21%)	7 (17%)	12 (25%)	
Increased	68 (77%)	33 (80%)	35 (73%)	
Change in feelings of isolation				0.05
Decreased	2 (2%)	2 (5%)	0 (0%)	
No effect	16 (18%)	4 (10%)	12 (25%)	
Increased	71 (80%)	35 (85%)	36 (75%)	
Change in intrusive eating disorder thoughts				0.26
Decreased	6 (7%)	4 (10%)	2 (4%)	
No effect	17 (19%)	5 (12%)	12 (25%)	
Increased	66 (74%)	32 (78%)	34 (71%)	

In bivariate analysis, motivation to recover (*p* = 0.27), feelings of depression (*p* = 0.19), anxiety (*p* = 0.72), and intrusive ED thoughts (*p* = 0.26) were not associated with reported COVID-19 related familial economic disruptions, while feelings of isolation were borderline associated (*p* = 0.05) (Table 2). Within the participants with AN and AAN diagnoses only, 31% of participants indicated a similar decrease in motivation to recover from an ED (*p* = 0.50), 80% reported increased feelings of depression (*p* = 0.52), 83% reported increased feelings of anxiety (*p* = 0.52), 83% reported increased feelings of anxiety (*p* = 0.81), 85% reported increased feelings of isolation (*p* = 0.11), and 83% reported increased feelings of intrusive ED thoughts (*p* = 0.74).

Participants reporting any familial economic disruptions were found to have nearly twice higher odds of feelings of depression (*p* = 0.30), anxiety (*p* = 0.41), isolation (*p* = 0.23), and intrusive ED thoughts (*p* = 0.36) and decreased motivation to recover from an ED (*p* = 0.17) than those reporting no familial disruptions after adjusting for age and restrictive diagnosis (Table 3), though these findings were not statistically significant. Results also found similar findings for the subset of participants diagnosed with AN/AAN with those reporting any familial economic disruption having higher odds of feelings of depression (aOR = 1.33 (95% CI 0.41 to 4.28), *p* = 0.63), anxiety (aOR = 1.51 (95% CI 0.42 to 5.42), *p* = 0.53), isolation (aOR = 1.54 (95% CI 0.41 to 5.79); *p* = 0.52), and intrusive ED thoughts aOR = 1.45 (95% CI 0.42 to 5.09); *p* = 0.56) and decreased motivation to recover from an ED (aOR = 1.47 (95% CI 0.54–3.97); *p* = 0.45) relative to those reporting no familial disruptions after adjusting for age and restrictive diagnosis.

**Table 3:** Unadjusted/adjusted association between self-reported pandemic-related economic consequences and worsening of eating disorder/mental health symptoms (*n* = 89)

Outcome	Unadjusted		Adjusted^b^	
	odds ratio (95% CI)^a^	*p*-value	odds ratio (95% CI)^a^	*p*-value
Change in motivation to recover from eating disorder	1.94 (0.77, 4.90)	0.16	1.93 (0.76, 4.92)	0.17
Change in feelings of depression	1.62 (0.62, 4.22)	0.33	1.76 (0.61, 5.07)	0.30
Change in feelings of anxiety	1.53 (0.56, 4.17)	0.40	1.60 (0.52, 4.89)	0.41
Change in feelings of isolation	1.94 (0.66, 5.75)	0.23	2.07 (0.64, 6.74)	0.23
Change in intrusive eating disorder thoughts	1.46 (0.56, 3.85)	0.44	1.68 (0.55, 5.12)	0.36

^a^ Odds ratios reported are for the primary predictor impact on family (any vs. none) predicting a worsening for each outcome (decreased vs. no change/increased for motivation to recover; increased vs. no change/decreased for feelings of depression, anxiety, isolation and intrusive eating disorder thoughts)

^b^ Adjusted for age and restrictive diagnosis

*CI* Confidence interval

## Discussion

This study examined the association between COVID-19 related familial economic disruption and ED/MH concerns in adolescent and young adults seeking treatment for EDs, five months into the enactment of state-level mandates around social distancing. Our participants reported a high degree of impact from the pandemic with many reporting economic impact to their families and large percentages reporting increasing MH and ED specific symptoms. Those who reported economic impacts of the pandemic had higher odds of reporting MH and ED-specific symptoms, yet these results were not significant.

These findings are in line with Guessoum et al.’s [16 finding that adolescent patients with EDs are vulnerable during COVID-19-related lockdown measures. [13] Research suggests that the COVID-19 pandemic increases ED symptomology due to disruption to daily activities and increased exposure to ED triggering content which results in compensatory exercising during lockdown, coupled with lower protective capacity from a lack of social support, and access to care [16, 17]. Prior work has also indicated that the COVID-19 pandemic has worsened parent and child MH outcomes, particularly in families experiencing economic hardships associated with job and income loss, caregiver burden and familial illness [18]. Given that this study was cross-sectional, we were not able to suggest causality of economic familial disruptions with MH and ED concerns, and prior work has alluded to the bidirectional relationship of those outcomes [23, 26, 27].

We suggest that the pandemic would have greater negative consequences on individuals with pre-existing mental health conditions, such as EDs, as alluded to in systematic reviews on the mental health effects of those with underlying mental health symptomology [23–25]. This trend may also be found for those who are essential personnel and individuals with family members who were essential personnel and medical professionals who had to work longer hours on the job [3].

While our results found a large percentage of our study population self-reported increased MH and ED-related symptoms that they attributed to the pandemic, our results did not support our hypothesis that economic disruption would increase the likelihood of reporting worsening MH and ED symptoms. We note that our study uses binary categorization of self-reported data on whether participants faced economic familial disruption or not with just under half reporting some economic disruption, thereby limiting generalizability. Our post-hoc power analyses found that our lower response rate contributed to low power to detect a statistically significant differences between those with and without reported economic consequences in motivation to recover from ED (29% power), intrusive thoughts about ED (13% power) changes in feelings of depression (17% power), anxiety (13% power), isolation (9% power). Our wide confidence intervals also suggest that this lack of statistical significance is likely due to low power to detect significant associations between pandemic-related economic consequences and ED/MH concerns as well as motivation to recover.

Due to the moderate response rate of our study sample, and the majority of the RECOVERY study participants identifying as White and have self-reported restrictive ED, we acknowledge that our findings may not be generalizable to a broader population of individuals with EDs. Our sample size also prevented us from stratifying our results by ED diagnosis. To this end, we were only able to compare individuals who were diagnosed with AN to those with other EDs (ARFID, BN, or BED) in our sample and cannot make conclusive findings on the differential influences of familial economic support on negative MH/ED concerns in patients diagnosed other types of EDs. However, our sensitivity analysis for only participants diagnosed with AN/AAN indicated that findings were similar for those diagnosed with AN/AAN compared to the larger participant sample.

Our small subsample of the RECOVERY cohort that chose to take part may represent those who found the pandemic particularly difficult and therefore, were likely to respond to this off-cycle survey or could represent those less affected and thus, more able to respond. [3] Perhaps an additional factor was our inability to offer remuneration for completion of this COVID-19 survey unlike our other surveys. Furthermore, our participant sample of RECOVERY cohort participants were engaged in clinical care, which may result in better perception of ED/MH concerns and higher motivation to recover than those not seeking or able to access clinical care during the pandemic.

A survey conducted by the Center on Budget and Policy Priorities indicated that in October 2021, nearly 20 million individuals lived in households with not enough to eat and 12 million behind rent [19]. This economic fallout was particularly present among Black and Latinx and other communities of color. In a study by Patrick et al. (2020), researchers found that COVID-19 has led to 50% of families having lost childcare, 11% reporting worsening food insecurity, and 16% reporting changes in insurance status [20]. Han and Hart (2022) illustrated that within a parents sample, certain elements of job precarity, namely vulnerability at work, low material reward, and job loss during the COVID-19 pandemic was associated with higher aggravations in parenting and lower child happiness [21]. Our findings of high degree of economic hardship and high degree of worsening MH and ED symptoms are in concert with these findings.

The pandemic has underscored the need for building resilient systems of support and health services to respond to global economic disruptions. [28] Suggestions from prior research indicate that for such to occur local preparedness and response is key. This can be done through the strengthening of digital, internet-based technologies to maintain balances in healthcare and education systems for adolescents and young adults, through specialized outpatient care teams, and through effective leadership and organizational support in hospitals [29–31]. Additionally, by stronger routine involvement of patients and families in care, disrupted families will be better equipped to manage stressful circumstances such as a global pandemic [32].

Taking these findings along with our study, future studies should aim to develop a more comprehensive understanding of the pandemic-related economic burdens experienced by families and patients with EDs, so that future prevention and treatment interventions effectively target such burdens for both, patients and caregivers. Furthermore, future studies could examine how economic familial disruptions have affected ED-related concerns and motivation to recover from an ED over time in a larger participant sample, along with how these disruptions may hinder help-seeking patterns for ED treatment. Along this vein, future work can also look into economic changes such as financial precarity and uncertainty, coupled with social isolation and job loss, in familial relationships at large and their impact on youth with ED [33–35].

## Conclusion

Given trends showing that patients with EDs reporting familial economic disruptions because of the pandemic have worsening ED/MH-related concerns and motivation to recover, future studies should explore this association in larger study samples. Clinicians should consider these associations when helping patients to move toward recovery during COVID-19.

## Data Availability

The datasets generated and/or analyzed during the current study are not publicly available due to patient confidentiality and the commitment given to all participants in protecting their identity. Data are available de-identified from the corresponding author on reasonable request and IRB approval.

## Abbreviations

ED: Eating disorder
MH: Mental health
AYA: Adolescent/Young adult
AN: Anorexia nervosa
AAN: Atypical anorexia nervosa
ARFID: Avoidant restrictive food intake disorder
